# Seed‐Mediated Growth and Advanced Characterization of Chiral Gold Nanorods

**DOI:** 10.1002/adma.202412473

**Published:** 2024-10-09

**Authors:** Bing Ni, Guillermo González‐Rubio, Kyle Van Gordon, Sara Bals, Nicholas A. Kotov, Luis M. Liz‐Marzán

**Affiliations:** ^1^ Department of Chemical Engineering University of Michigan 2800 Plymouth Road Ann Arbor Michigan 48109 USA; ^2^ College of Chemistry Beijing Normal University Beijing 100875 China; ^3^ Departamento de Química Física Universidad Complutense de Madrid Avenida Complutense s/n Madrid 28040 Spain; ^4^ CIC biomaGUNE Basque Research and Technology Alliance (BRTA) Paseo de Miramón 194 Donostia‐San Sebastián 20014 Spain; ^5^ Electron Microscopy for Materials Science (EMAT) and NANOlab Center of Excellence University of Antwerp Groenenborgerlaan 171 Antwerp 2020 Belgium; ^6^ Ikerbasque Basque Foundation for Science Bilbao 48009 Spain; ^7^ Biomedical Research Networking Center Bioengineering Biomaterials and Nanomedicine CIBER‐BBN Paseo de Miramón 194 Donostia‐San Sebastián 20014 Spain; ^8^ Cinbio Universidade de Vigo Campus Universitario s/n Vigo 36310 Spain

**Keywords:** chiral gold nanorods, chirality measures, electron tomography, helicity, seed‐mediated growth

## Abstract

The controlled growth of gold nanostructures with complex shapes and reduced symmetry, exemplified by chiral gold nanorods and nanoparticles, is one of the most dynamic fields of nanochemistry. A timely summary of underlying concepts, including growth mechanisms and redefined chirality measures, would further promote this research area. In this perspective, we aim to establish qualitative connections between the chiral shapes and growth conditions, specifically for the seed‐mediated synthesis of chiral gold nanorods as a convenient case of chiral morphogenesis. The crystallographic and morphological features of achiral nanorods used as seeds, the experimental conditions during chiral growth, and the symmetry of the chiral inducers, can all be exploited to obtain nanorods with intricate chiral shapes. Chirality characterization (such as electron tomography techniques) and quantification (including chirality measures) emerge as critical aspects to comprehensively explore and understand such structures, enabling optimization of their geometric and optical features. We conclude by discussing relevant challenges to be addressed toward a better controlled synthesis of chiral plasmonic nanostructures.

## Graphical abstract

Recent progress in bottom‐up wet‐chemical synthesis of gold nanoparticles has created remarkable opportunities to produce nanoparticles with well‐defined chiral shapes and strong chiroptical activities. This perspective focuses on the seed‐mediated synthesis of chiral gold nanorods as a convenient case of chiral morphogenesis. Growth mechanisms, chirality measures, and advanced electron microscopy characterization techniques are discussed, aiming to connect chiral morphologies, growth conditions, and optical behavior.

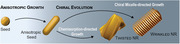

## 1 Introduction

The ability to control morphology at the nanometer scale drives the rapid development of nanoscience and nanotechnology. After decades of thorough investigation of highly symmetric nanoparticles (NPs), including spheroids or rods, increasing attention is currently being paid to reduced symmetries at the nanoscale, including chiral morphologies.^[^ ^1^, ^2^, ^3^, ^4^ ^]^ One can also point out that it is a scientific progression of simple to complex nanoscale structures with more sophisticated properties that require much finer elaboration of reaction conditions than for spherical NPs. Precise design and synthesis of chiral NPs provide the foundation for subsequent exploration of unusual properties combining structural features from inorganic and biological chemistry,^[^ ^5^ ^]^ which are expected to bring not only new concepts in chemistry but also new solutions to existing challenges, such as nanoscale biomimetics,^[^ ^5^, ^6^ ^]^ enantioselective catalysis,^[^ ^7^ ^]^ optics,^[^ ^8^, ^9^ ^]^ biosensing,^[^ ^10^ ^]^ and even scalable 3D printing of nanoscale structures, among others.^[^ ^11^ ^]^ Reliable methods for chiral NP growth are, therefore, key to unlocking the potential of inorganic nano‐stereochemistry.

Among a wide variety of colloidal systems, Au NPs attracted attention already in the 19^th^ and 20^th^ centuries, and allowed fundamental discoveries related to the growth and behavior of colloidal particles with high symmetry.^[^ ^12^, ^13^, ^14^, ^15^, ^16^ ^]^ Today, they offer a fertile playground for the synthesis of Au NPs with reduced symmetry and chiral features, including dissymmetric and asymmetric nanostructures.^[^ ^3^, ^6^, ^17^, ^18^ ^]^ The term “dissymmetric” was used in French crystallography literature over 200 years ago, to define crystals “lacking certain symmetry properties relative to the crystal axis”.^[^ ^19^ ^]^ In most cases, chiral Au NPs possess rotation axes and can, therefore, be classified as dissymmetric structures rather than asymmetric (a terminology typically used in small molecule stereochemistry, meaning “lacking all symmetry elements”). However, the removal of symmetry elements in nanocolloids from gold is challenging, primarily because of the highly symmetric face‐centered cubic (fcc) Au crystalline lattice (space group Fm $\bar{3}$ m), which thermodynamically favors the growth of symmetric morphologies (e.g., NPs with the same point group as the lattice).^[^ ^20^, ^21^, ^22^, ^23^ ^]^ Albeit possible, lowering the symmetry of Au NPs in a controlled manner (especially in a colloidal system) is, therefore, far from straightforward. For this reason, perhaps the first observations of giant optical dissymmetry in plasmonic materials were made in chiral assemblies of achiral Au NPs – superstructures where the particles remain spheroidal but the overall nanostructure is dissymmetric.^[^ ^24^ ^]^ Since the first demonstration of discrete chiral Au helicoids, seed‐mediated growth of dissymmetric NPs has been rapidly developing for various shapes of Au seeds. Among them, many efforts have been paid to Au nanorods (NRs), where their distinctive anisotropy can lead to unique chiral structures not obtained using more symmetric nanoparticles as seeds. These efforts led to a fundamental understanding of crystal growth mechanisms and versatile practical applications ascribed to the tunability of Au NR plasmon bands, from the visible to the near IR (NIR).^[^ ^25^ ^]^ Nowadays, the use of Au NRs offers unmatched control over the growth of dissymmetric NPs with tailored chiral plasmon bands, in turn enabling the fabrication of advanced chiroptically active nanomaterials.

In this scenario, where the field of dissymmetric synthesis is evolving rapidly, sufficient elements have been reported that allow us to discuss the mechanisms underlying the chiral growth of Au NPs, with a focus on the particular case of chiral Au NRs. Without losing generality, we start with a brief introduction to the main concepts related to the seed‐mediated growth of dissymmetric NPs, the importance of mirror plane removal, and the kinetic nature of chiral NP products and their growth process. With this general picture in mind, we proceed to the discussion of the most relevant approaches exploited for directing chiral growth on Au NRs, namely: chemisorption‐directed growth and micelle template‐directed growth, where the relationship between the growth conditions and the chiroptical properties of the products are qualitatively discussed. Since the morphologies of chiral Au NRs can be complex, recent advancements in electron tomography techniques and data analysis methods (including chirality measures) have become an unprecedented tool for the quantitative investigation of subtle chiral features, toward understanding and optimizing dissymmetric growth. The final section explores prospects and potential applications of seed‐mediated dissymmetric synthesis, based on the concepts and ideas discussed herein.

## 2 General Concepts in Dissymmetric Gold Nanoparticle Growth

### 2.1 Seed‐Mediated Growth of Dissymmetric Nanoparticles

**Figure 1:**
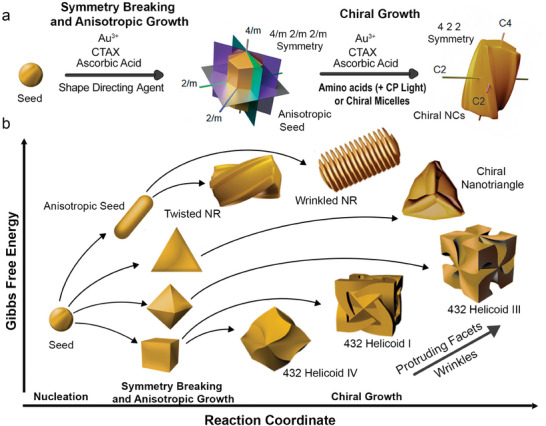
a) Schematic view of the seed‐mediated synthesis of chiral Au NRs from anisotropic seeds. During symmetry breaking and anisotropic growth, isotropic seeds evolve into faceted NRs, which are subsequently used as seeds to grow chiral NRs. Adapted with permission from ref. [25] (Copyright 2023 The Authors). b) Possible scenarios for the seed‐mediated growth of single‐crystal (cube, octahedra, nanorods) and monotwinned (nanotriangle) seeds into chiral NPs with different types of anisotropy, protruding facets and wrinkles, and related Gibbs free energies. Au NRs can evolve into either twisted or wrinkled NRs, octahedra into 432 Helicoid III NPs, mono‐twinned nanotriangles into triangles with protrusions resembling propeller blades, whereas chiral growth of nanocubes leads to 432 helicoids I and IV. Twisted and wrinkled NRs, triangles with protrusions resembling propeller blades, 432 helicoids I and IV, and 432 helicoid III models are adapted with permission from refs. [6, 17, 26, 35], respectively (Copyright 2023 The authors, Copyright 2020 The Authors, Copyright 2022 The Authors, and Copyright 2018 Nature Publishing Group, in order).

Engineering of chiral Au NPs has been long recognized as an extraordinary challenge by the scientific community, and a great deal of research is still focused on their synthesis and characterization. Colloid chemistry routes offer unmatched control over the morphology and chiroptical activity of Au NPs. These methods typically rely on the spatiotemporal separation of nucleation and growth, to tailor seed crystallinity and eventually govern NP size and morphology.^[^ ^6^, ^17^, ^18^, ^25^, ^26^, ^27^, ^28^, ^29^, ^30^, ^31^, ^32^, ^33^, ^34^, ^35^ ^]^ Therefore, we first provide a general picture of evolution of nanoscale chiral shapes. The so‐called seed‐mediated synthesis of chiral Au NPs involves three fundamental steps: i) nucleation of gold atoms into isotropic NPs, ii) reduction of NP symmetry to form anisotropic seeds (i.e., NPs with defined shapes on which chiral growth is induced) with selected morphology and exposed facets through deposition of additional Au atoms, and iii) removal of mirror‐plane symmetry elements via dissymmetric growth (**Figure** **1**). The nucleation step helps avoid secondary nucleation, which would lead to broad size distributions. More importantly, it allows tuning seed dimensions and crystal habits. The latter, ascribed to the number of planar defects (i.e., single crystal, mono‐twinned, or penta‐twinned), can critically determine the symmetry of the anisotropic seeds^[^ ^21^, ^36^ ^]^ and, consequently, the chirality of the resulting Au NPs.^[^ ^6^, ^30^, ^31^, ^32^, ^33^ ^]^ In the symmetry breaking and anisotropic growth stages, one aims to tailor the anisotropy, size, and exposed facets of Au NPs, which will direct the evolution of chiral shapes.^[^ ^17^, ^18^, ^32^, ^33^ ^]^ Finally, the dissymmetric growth stage is intended to define the morphology, dimensions, and optical activity of chiral features in Au NPs. In general, the three stages outlined above are separated in both time and space. However, examples can be found where the symmetry breaking and dissymmetric growth steps are confined to the same growth solution.^[^ ^28^, ^34^ ^]^

### 2.2 Mirror Plane Removal

Great progress in seed‐mediated routes has been achieved during the past two decades, leading to various anisotropic morphologies, such as nanocubes, octahedra, nanotriangles, or NRs, with tailored dimensions.^[^ ^21^, ^37^, ^38^, ^39^, ^40^, ^41^ ^]^ These syntheses generally involve the use of HAuCl_4_ as gold precursor, long‐alkyl‐chain quaternary ammonium halide surfactants (most frequently cetyltrimethylammonium halides, CTAX, where X = Cl or Br), and ascorbic acid as a mild reducing agent. Shape‐directing agents are also often required for anisotropic growth, such as Ag^+^ or I^−^ ions in the synthesis of NRs^[^ ^42^ ^]^ and nanotriangles,^[^ ^38^ ^]^ respectively.^[^ ^43^ ^]^ Shape‐directing agents can modify the NP's free surface energy, thereby enabling the stabilization of unfavorable facets that typically emerge during anisotropic growth, thus sustaining an anisotropic shape in the final NP. Notably, most of the reported chiral Au NP synthesis routes involve HAuCl_4,_ CTAX, and ascorbic acid, as well as amino acids and peptides or chiral co‐surfactants (i.e., chiral micelles) as chiral shape‐directing agents (also called chiral inducers). Such molecules can facilitate the removal of mirror planes in the anisotropic seeds but may preserve rotational symmetry to some extent. For example, in the case of single‐crystalline Au NR seeds, with a 4/mmm symmetry, removal of mirror planes may lead to the formation of twisted nanorods with 422 symmetry (Figure 1). By taking advantage of the photothermal effect and hot carrier distribution, irradiation with circularly polarized light can also effectively enhance the effect of chiral inducers.^[^ ^6^, ^44^, ^45^, ^46^, ^47^, ^48^, ^49^, ^50^ ^]^ Some typical dissymmetric NP synthesis protocols are summarized in **Table** **1** and Table S1, Supporting Information.

**Table 1:** Typical seed‐mediated dissymmetric growth conditions for chiral Au NPs.

		Chiral inducer		HAuCl_4_ (mM)	[AA] / [HAuCl_4_]				
Chiral morphology	Seed	Type	Conc. (µM)			Surfactant	Max.|g‐factor|	Note	Refs.
432 helicoid I	Cube	Cys	0.092	0.37	23.8	CTAB	0.03 (565 nm)		[17]
432 helicoid III	Octahedron	GSH	4.6	0.37	23.8	CTAB	0.2 (620 nm)		
422 twisted rod	Single‐crystalline rod	Cys	0.074	0.0067	1600	CTAC	0.106 (650 nm)	9 HAuCl_4_ additions at varied temperatures to enhance chirality	[25]
Twisted rod with fivefold rotational symmetry	Elongated penta‐twinned decahedron	GSH	≈19–47	0.63	5	CTAB	0.06 (650 nm)	KI is added to modify the chiral morphology	[31]
Rod with spiral wrinkles	Single‐crystalline rod	BIAMINE	2500	0.5	317	CTAC	0.2 (1100 nm, 1400 nm)	Chiral inducer serves as a co‐surfactant to form chiral micelles	[18]
422 twisted rod	Single‐crystalline rod	LipoCYS	20	0.19	3684	CTAC	0.025 (620 nm)	Concentration of chiral inducer can tune the growth mode	[35]
Twisted rod with wrinkles			45				0.015 (700 nm)		
Rod with spiral wrinkles			90				0.066 (720 nm)		
Propeller‐like chiral particle	Triangular plate	CYP	3.7	0.37	9.5	CTAB	0.44 (727 nm)	Circularly polarized light is introduced to assist chiral growth	[6]

Conc.: concentration; AA: ascorbic acid; Cys: cysteine; GSH: glutathione; BINAMINE: 1,1′‐binaphthyl‐2,2′‐diamine; LipoCYS: 2‐amino‐N‐decyl‐3‐mercaptopropanamide; CYP: cysteine–phenylalanine.

### 2.3 Chiral Nanoparticles as Kinetic Products

An essential consideration to understand the formation of dissymmetric NPs is the emergence of chiral protruding facets and wrinkles during the growth process. Chiral NPs often have rough surfaces and sharp features enclosed by high‐energy (high‐index) facets.^[^ ^17^, ^18^, ^51^ ^]^ The expected Gibbs free energy of chiral systems is, therefore, larger than that of a spherical isochoric NP with smooth surfaces (i.e., minimized area and lower surface‐to‐volume ratio). One can thus identify chiral NPs as kinetic or out‐of‐equilibrium products from a thermodynamic standpoint. Indeed, any kind of symmetry reduction and dissymmetric evolution is systematically accompanied by an increase in the Gibbs free energy of the NP.^[^ ^20^, ^36^ ^]^ In this respect, to promote the formation of out‐of‐equilibrium chiral NPs, two phenomena ought to take place: i) kinetics‐promoted emergence of protruding facets or wrinkles via asymmetric deposition of gold atoms; ii) stabilization of these features, thereby amplifying dissymmetric growth. The first effect can occur when the rate at which new gold atoms are added to the growing crystal is higher than the speed at which they diffuse on its surface. In general, deposition occurs at sites with higher curvature, such as corners, protruding facets, or wrinkles. These sites are more reactive due to their sharp features, which can also reduce the steric barrier of gold precursor deposition due to the reduced surface ligand coverage. Deposition rates can be experimentally enhanced by increasing the concentration of the reducing agent (usually ascorbic acid) to boost the gold precursor reduction rate or by decreasing the surfactant concentration. In fact, the [ascorbic acid]:[HAuCl_4_] ratio used during the growth of Au NPs with high optical activity is, in most cases, above 20 and can go up to ≈3600 (Table 1).^[^ ^17^, ^18^, ^25^, ^26^, ^28^, ^31^, ^32^, ^33^, ^34^, ^35^ ^]^ These values represent between ≈12‐ and ≈2250‐fold (Table S1, Supporting Information) increments over the [ascorbic acid]:[HAuCl_4_] ratios typically used in achiral Au NR synthesis (e.g., [ascorbic acid]:[HAuCl_4_] ratio of 1.6).^[^ ^41^ ^]^ On the other hand, the stabilization of emergent chiral features demands surface ligands that can preferentially chemisorb and passivate the exposed high‐index facets.^[^ ^17^, ^32^, ^35^, ^51^, ^52^ ^]^ In what follows, we discuss various strategies based on the nature of chiral inducers that have been used to promote enantioselective growth of Au NPs and Au NRs.

## 3 Chemisorption‐Directed Growth of Chiral Gold Nanorods

This strategy relies on the use of thiolated peptides or amino acids as chiral inducers, owing to their ability to strongly passivate Au surfaces and promote enantioselective growth of chiral facets. This effect has been ascribed to two main factors: i) their chiral configuration and ii) the presence of a thiol group.^[^ ^53^ ^]^ First demonstrated by Nam and co‐workers in 2018,^[^ ^17^ ^]^ using cubic seeds and cysteine or glutathione as chiral inducers, the chemisorption strategy is arguably the most explored one to date. According to density functional theory (DFT) calculations, cysteine moieties can interact differently with various Au surfaces in a three‐point contact manner.^[^ ^54^, ^55^, ^56^ ^]^ As a result, strong enantioselective passivating effects would be promoted, leading not only to dissymmetric NP evolution but also to stabilization of protruding facets (Figure 1). Note the origin of chirality in Au NPs is different than that in small Au clusters^[^ ^57^ ^]^ and many other nanomaterials.^[^ ^58^ ^]^ In the latter, a distortion of the crystal lattice takes place, which has profound differences both in optical and chemical properties.

**Figure 2:**
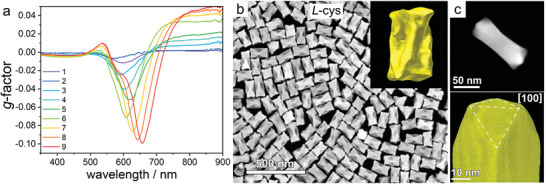
a) Evolution of the optical activity of Au NRs during the growth of chiral features via successive HAuCl_4_ additions in the presence of L‐cys. b) High‐angle annular dark‐field scanning transmission electron microscopy (HAADF‐STEM) image showing the twisted morphology resulting from Au NR overgrowth after nine successive HAuCl_4_ additions. Inset: single chiral Au NR electron tomography reconstruction. c) HAADF‐STEM image (top) and visualization of the 3D reconstruction (bottom) of an Au NR obtained after one chiral growth step. The white dashed lines indicate the location of {520} facets. Scale bar: 10 nm. Adapted with permission from ref. [25] (Copyright 2023 The Authors).

Due to large energy gain for the formation of gold crystal lattice in the standard highly symmetric form, the emergence of protruding facets is experimentally far from straightforward. A relevant example is the case of twisted single‐crystalline Au NRs, where a repetitive growth method was required to obtain chiral features in a reproducible manner^[^ ^25^ ^]^ (**Figure** **2**a,b). In this case, it was proposed that single‐crystalline NR seeds initially evolved into intermediate structures with four achiral {520} facets at the tips, which would act as precursors for the subsequent emergence of chiral {521} facets under the influence of cysteine. Consequently, the surface areas of ***S***‐type surfaces will gradually become larger than those of ***R***‐type surfaces during growth, leading to the removal of mirror planes. Since the rotational symmetry is not influenced by the unequal ***R/S***‐type surfaces, it can be maintained to some extent and contribute to the emergence of helical structures. Thereby, the proposed process could eventually reduce the NR symmetry from 4/mmm to 422, that is, into a chiral twisted NR (Figures 1a and 2c). Nonetheless, it is still unclear how the enantioselective growth of atomic chiral facets can be connected to the nanoscale handedness of the twisting structures, with complicated protruding sites and rough surfaces.

In the chemisorption‐directed growth model, the concentration of chiral inducer, typically ranging from nM to µM (Table S1, Supporting Information), is another critical aspect, and minor variations may lead to dramatic changes in the chiral features of the resulting dissymmetric NPs. For example, in the synthesis of twisted Au NRs,^[^ ^25^ ^]^ higher cysteine concentrations led to increased surface roughness and reduced chiroptical activity. The reason behind this effect was attributed to the fact that enantioselective surface passivation decreases for increased coverage of gold surfaces with cysteine. Similar effects have been reported for other systems, even showing a reversal of the optical activity for different chiral inducer concentrations.^[^ ^48^ ^]^

**Figure 3:**
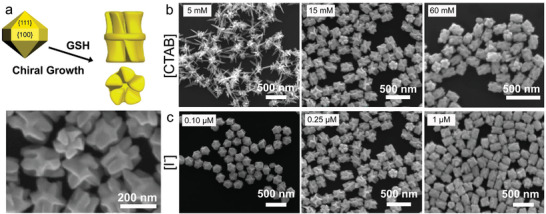
a) Schematics (top) showing the growth of a left‐handed penta‐twinned Au NR from elongated decahedra, using glutathione (GSH) as chiral directing agent and a representative SEM image of the synthesized chiral Au NRs (bottom). b,c) SEM images of penta‐twinned chiral Au NRs obtained in the presence of increasing concentrations of CTAB (b) and I^−^ (c). Reproduced with permission from ref. [31] (Copyright 2023 The Authors).

Another experimental parameter that needs consideration is the concentration and nature of halides present during chiral growth. Halide ions show different affinity for different gold facets, thereby influencing the thermodynamic aspects of morphological evolution.^[^ ^20^, ^36^, ^59^ ^]^ Additionally, the formation of complexes (X = Cl, Br, I) modifies the reduction potential of Au ions (Cl > Br > I), directly impacting growth kinetics.^[^ ^60^, ^61^ ^]^ In recent examples,^[^ ^31^, ^62^ ^]^ using glutathione as a chiral inducer and elongated penta‐twinned decahedra as seeds, well‐defined fivefold NRs with chiral structure were obtained by optimizing the concentrations of iodide and CTAB (highest *g*‐factors were obtained at 0.25 µM and 15 mM of iodide and CTAB, respectively, (**Figure** **3**). For example, low concentrations of CTAB (5 mM) favored the growth of spiky NPs with low chiroptical activity, whereas the presence of CTAB at high concentrations (60 mM) induced the formation of NPs with slightly less defined chiral features (Figure 3b).^[^ ^63^ ^]^ At low I^−^ concentration (0.10 µM), the lateral growth of chiral NRs was reduced, but protruding facets were formed. However, at higher I^−^ concentrations (1 µM), growth into chiral morphologies was significantly reduced (Figure 3c). This effect was ascribed to passivation of {111} facets, which seem to play an active role in the emergence of chiral branches.

## 4 Chiral Micelle‐Directed Growth of Chiral Gold Nanorods

**Figure 4:**
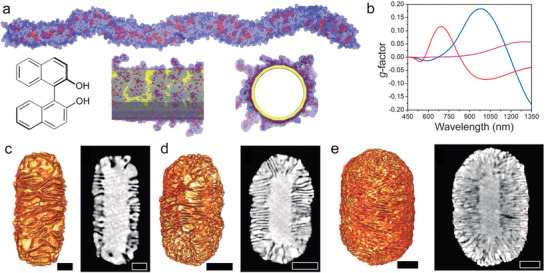
a) Molecular dynamics simulations of a helical micelle formed by BINOL and CTAC in water (top), and absorbed onto an Au cylinder (bottom). b–e) Spectral evolution of the anisotropy factor (b), electron tomography reconstructions (c, d, e‐left panels), and selected orthoslices (c, d, e‐right panels) of chiral Au NRs with increasing chiral shell thickness: 22 nm (red spectrum), 41.5 nm (blue spectrum), and 73 nm (purple spectrum). Scale bars: 50 nm. Reproduced with permission from ref. [18] (Copyright 2020 The American Association for the Advancement of Science).

As discussed in Sections 2 and 3, most chiral NP synthesis methods rely on the use of CTAX (X = Cl, Br) as the surfactants that provide colloidal stability of nanostructures. However, when molecules with axial chirality, such as 1,1′‐Bi‐2‐naphthol (BINOL, a chiral co‐surfactant), enter CTAX micellar aggregates, the resulting mixed micelles can also induce the growth of chiral features.^[^ ^64^ ^]^ On the basis of this concept, Liz‐Marzán, Bals, and colleagues proposed the use of chiral micelles to template the chiral growth of Au NRs.^[^ ^18^ ^]^ The designed methodology, referred to as “chiral‐micelle templating” or “micelle‐directed chiral growth”, involves the co‐assembly of chiral co‐surfactants with cetyltrimethylammonium chloride (CTAC) to form micelles with a helical structure.^[^ ^64^ ^]^ This process might occur via a “sergeants and soldiers” mechanism, with the atropisomerism of BINOL or 1,1′‐binaphthyl‐2,2′‐diamine (BINAMINE) directing the chiral arrangement of achiral molecules, which was confirmed by molecular dynamics (MD) simulations (**Figure** **4**a). In the presence of Au NRs, such chiral micelles would adsorb in a coiled conformation around the NR surface, serving as a chiral template during seeded growth, which resulted in the formation of distinct wrinkles with an evident directionality. Importantly, this growth was remarkably uniform across the entire particle population for a given colloid preparation. The use of BINAMINE rather than BINOL was crucial to achieve efficient chirality transfer, as indicated by strong chiroptical signals in the final product. The presence of amine moieties in BINAMINE (compared to hydroxyls in BINOL), with a greater affinity for gold surfaces, was proposed to result in a more stable helical template. Through the interplay between the NR's aspect ratio and the thickness of the chiral shell (controlled by the concentration ratio between gold seeds and Au^3+^), tunable chiral plasmon modes within the visible and the near‐infrared, and high dissymmetry factors (≈0.20) were obtained (Figure 4b–e).^[^ ^18^ ^]^ In this case, the formation of thin and extended wrinkles was likely determined by the fast reduction kinetics obtained by using a high [ascorbic acid]:[HAuCl_4_] ratio of 320 (Table 1).^[^ ^65^ ^]^

Despite the high *g*‐factors obtained via the chiral micellar template strategy, an important observation was the limited stability of the wrinkled NRs. The intricate structure of the thin and extended wrinkle network possesses a high surface area and prominent curvatures. To reduce the unfavorable surface energy, the system tends to evolve toward a more thermodynamically favored conformation where wrinkles are removed. In the initial study,^[^ ^18^ ^]^ a significant decay of the wrinkle NR chiroptical activity was observed 2 weeks after synthesis, and electron tomography characterization revealed almost complete surface smoothening. Similar reshaping effects have been previously reported for nanostars, nanotriangles, and NRs, usually ascribed to surface diffusion phenomena: surface atoms located at high curvature sites and poor coordination environments, diffuse and rearrange to reach a more thermodynamically favored conformation, often promoted by bromide and iodide ions. To overcome the long‐term structural stability issue, solvent exchange into a low concentration of CTAC can be used. Alternatively, coverage with a polyisobutylene‐alt‐maleic anhydride shell has been shown to impart better protection against heating and corrosives.^[^ ^65^ ^]^ A recent study also found that Na_2_S treatment of chiral particles significantly improved their stability.^[^ ^66^ ^]^

## 5 Chirality Measures and Advanced Electron Microscopy to Interrogate Chiral Morphologies

Chirality measures are used to quantify the dissymmetry of NPs, which is useful to understand their structure and to eventually monitor chiral evolution, or for quantitative analysis of structure‐property relationships. Chirality quantification has often been based on the material's optical properties, using the dissymmetry factor (*g*‐factor) to characterize the strength of optical activity in a quantitative manner. However, this method might not account for geometric dissymmetry. During the development of stereochemistry, several methods have been proposed to address geometrical aspects for small molecules, adopting the definition of chirality (i.e., the geometrical property for an object – or a spatial arrangement of points or atoms – of being non‐superimposable onto its mirror image)^[^ ^67^ ^]^ as a starting point. We summarize below the most common chirality measures.

i) Hausdorff Chirality Measure (HCM). This measure is based on a mathematical method where the chiral molecule or object is abstracted as a set of points in an *n*‐dimensional space.^[^ ^68^ ^]^ In the following step, the minimum Hausdorff distance between the object and its mirrored object is calculated. This implies that the object and its mirror image are imaginarily superimposed to achieve maximum overlap, and then the non‐superposable part is normalized to obtain a chirality index (without a sign).
ii) Continuous Symmetry Measures (CSM). This quantification strategy relies on determining how “far” an object is away from its nearest achiral reference. This can be realized by adding ligands onto an achiral backbone to achieve a chiral molecule. Ligand specificity would then be assigned to each linkage, and indices would be calculated accordingly.^[^ ^69^ ^]^ Beyond this specific operation, CSM is developed by considering the symmetry of a molecule as a continuous structural property and the minimum distances that the vertices of a shape must move to obtain the nearest achiral symmetry point group.^[^ ^70^, ^71^ ^]^ An unsigned index can also be obtained in this case.
iii) Osipov, Pickup, and Dunmur (OPD) Chirality Measure. The HCM and CSM can quantify geometrical chirality but cannot differentiate an object from its enantiomer, that is, left‐handed and right‐handed objects would have the same measure. A chirality index that changes sign for each enantiomer and is zero for achiral objects was proposed by M. A. Osipov, B. T. Pickup, and D. A. Dunmur (abbreviated as OPD chirality measure).^[^ ^72^ ^]^ An intrinsic molecular chirality tensor is derived from optical activity theory and yields two universal chiral indices. The first one provides information about absolute chirality, whereas the second index corresponds to anisotropic chirality, that is, the degree of chirality in different spatial directions. Note that OPD has been theoretically predicted to display so‐called, “chiral zeros”, which render its values uncorrelated.^[^ ^73^ ^]^ As it has been recently shown,^[^ ^74^ ^]^ OPD quantification of chirality is path‐dependent and must pass through a “flat state” to be practically applicable. In this case it displays high correlation with the optical activity.
iv) Graph Theoretical Chirality (GTC).^[^ ^75^ ^]^ GTC calculates chirality for crystalline nanostructures that can be represented as graphs, with atoms as nodes and bonds as edges. By identifying differences in alternate paths through two nodes in a graph, the path differences can be represented as a torsion, which is related to the chirality of an object. The method can be applied to any chiral structures and has the advantages of retaining a specific sign – positive or negative depending on the handedness of the enantiomer. At the same time, it mitigates the problem of chiral zeros typical for OPD and other pseudoscalar measure, because it inherently defines the path of reconfiguration. Furthermore, GTC is particularly suitable for NPs due to their simple representation as graphs embedded in the Cartesian space.

**Figure 5:**
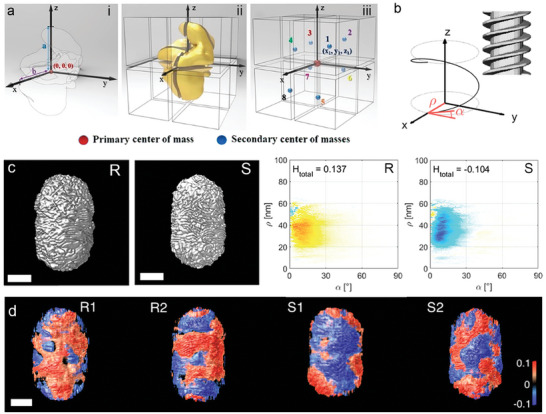
a) Schematic description of how a complicated chiral structure can be abstracted into eight coordinates. The center of mass of the NP is assigned as the primary center of mass. Then, the NP's farthest surface point is designated as the z‐axis, and the x‐axis is assigned as the farthest in the xy plane. The coordination system segments the NP into eight pieces, generating their centers of mass as the secondary centers of mass. The coordinates of the secondary centers of mass can be used for chirality measure calculations. Reproduced with permission from ref. [45] (Copyright 2019 American Chemical Society). b) A 3D model of a rod with a right‐handed helical shell, as an ideal example to illustrate helicity around a rod, where the local helicity can be calculated according to the radius (*ρ*) and inclination angle (*α*); c) Visualizations of the 3D electron tomography reconstructions of two Au NR enantiomers (left), and the corresponding helicity function H(*ρ*,*α*) plot (right) resulting in a total helicity H_total_. Scale bars are 50 nm. Reproduced with permission from ref. [78] (Copyright 2022 American Chemical Society). d) 3D‐color volume renderings of the helicity maps of wrinkled Au NRs synthesized in the presence of (*R*) or (*S*)‐BINAMINE. Blue colors indicate left‐handed helicity, and red colors indicate right‐handed helicity. Reproduced with permission from ref.[78] (Copyright 2022 American Chemical Society).

These chirality measures have been applied to chiral NPs,^[^ ^45^ ^]^ but are still sparsely explored, predominantly due to difficulties with computational approaches. Prior to chirality quantification, the 3D structure of a chiral NP must be faithfully captured. In this scenario, electron tomography has become an essential tool. After retrieving the precise 3D structure, different chirality measures can be applied. The above‐described HCM, CSM, and OPD were proposed to describe molecular chirality, thus involving systems comprising a reduced number of atoms with known coordinates and thus resulting in a mathematical problem that can be resolved at a computationally acceptable level. Conversely, NPs contain a much larger number of atoms. Therefore, considering all the atom positions during calculation of the traditional chirality measures poses a significant computational challenge. For this reason, reasonably abstracting a particle to a limited set of coordinates while preserving the chiral features is a solution to computing chirality measures for NPs. This can be achieved by partitioning the chiral NP so that the centers of the sub‐sections can be used to quantify chirality.^[^ ^45^ ^]^ For example, after an electron tomography measurement, the reconstructed chiral NP structure can be placed into a Cartesian coordinate system, with the center of mass of the particle placed at the origin. The direction of the coordinate axes is then placed according to the length of the particle. Next, the particle is sub‐sectioned into eight pieces by the coordinate axes, and the coordinates of their respective centers of mass can then be extracted for subsequent calculations of chirality measures (**Figure** **5**a). In the described example, Kotov and co‐workers showed that the calculated HCM, CSM, and OPD indexes correlated well with optical activity intensities; furthermore, the OPD index changed sign as the optical activity was reversed. A similar treatment was later applied to twisted bipyramids,^[^ ^6^ ^]^ where the calculated chirality indexes correlated well with biomedical and toxicological effects observed for specific NPs. Considering the problem of “chiral zeros” or more generally, the problem of the multiplicity of chiral harmonics,^[^ ^76^ ^]^ such a correlation becomes possible when there is a defined path for chiral reconfiguration that includes the achiral state.^[^ ^74^ ^]^

Besides that, there is an additional important difference between dissymmetry of simple molecules and nanostructures. For all chiral nanostructures prepared via the route of chiral inducers, chirality is observed at multiple scales.^[^ ^77^ ^]^ Prior to calculating the chirality measure, one needs to ask the question of what scale this chirality measure refers to. Kotov and co‐workers introduced a concept of chirality vector using an example of multiscale chirality in nanostructured bowtie‐shaped particles, for which the multiscale chirality was verified by multiple methods of spectroscopy and molecular dynamics simulations.^[^ ^74^ ^]^ The chirality vector was composed by chirality measures (such as OPD indexes) of molecular ligands, constituent nanoclusters, nanosheets, and overall bowties. An exponential relationship between chirality indexes and CD peak positions in the visible and NIR was observed for the OPD chirality measure at the largest scale but not at the molecular scale. These results illustrate the importance of using chirality measures to understand structure‐property relationships and to guide materials design, while advancing the fundamental concepts of chirality in chemistry.

An alternative approach consists of quantifying specific geometrical features, as recently proposed by Bals and co‐workers.^[^ ^78^, ^79^ ^]^ The helicity index, a pseudoscalar metric ranging from −1 to +1 for left‐ and right‐handed geometries, respectively, is calculated by integrating the inclination angles α (Figure 5b) of features over a part or the totality of a NR's surface. These inclination angles can be obtained by evaluating the directionality in the image gradients of cylindrical sections through an electron tomography reconstruction^[^ ^78^ ^]^ or by calculating the Euler angles of the normal vectors of surface elements in a triangulated mesh.^[^ ^79^ ^]^ The helicity index can thus be locally mapped to highlight the local handedness throughout the NP surface or computed over an entire NR to yield a single metric describing the overall handedness. Electron tomography characterizations and helicity calculation applied to study chiral wrinkled Au NRs revealed that areas with left‐ and right‐handed organization of wrinkles were present in every analyzed NR, regardless of which BINAMINE enantiomer was used during the synthesis (Figure 5c,d). Notwithstanding, an excess of one directionality was consistently identified, as dictated by the BINAMINE enantiomer, in agreement with the chiroptical behavior.^[^ ^80^ ^]^ This methodology was also exploited to investigate the growth mechanism of these particles, revealing the formation of an intermediate elongated octahedral structure (square cross‐section prism terminated in pointed tips), which was found to accommodate the adsorption of helical micelles more efficiently.^[^ ^65^ ^]^

**Figure 6:**
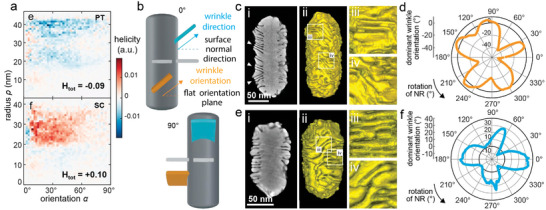
Characterization of chiral wrinkled Au NRs synthesized using penta‐twinned or single‐crystal Au NR seeds via the chiral micelle‐directed method. a) Helicity function plot for the chiral Au NRs obtained from penta‐twinned (PT) and single‐crystalline (SC) achiral Au NRs. b) Schematic illustration of the wrinkle direction angle (blue) and wrinkle orientation angle (orange). c–f) Central orthoslices (i) and isosurface visualization (i–iv) of the 3D reconstruction and enlarged graphs of chiral PT (c) and SC (e) NRs, respectively, showing the features of wrinkle direction angles and the corresponding polar plots (d, f) of the dominant wrinkle orientation a function of the rotation of each particle around its major axis (d: PT NRs; f: SC NR). Reproduced with permission from ref.[81] (Copyright 2024 The Authors).

More recently, electron tomography and helicity calculations were exploited to investigate an interesting phenomenon: the reversal of optical handedness when using penta‐twinned Au NRs as seeds instead of single‐crystalline Au NRs in the growth of wrinkled chiral Au NRs. Although the calculations revealed distinct helicities for the two types of chiral NRs (**Figure** **6**a), two definitions were introduced to improve the wrinkle geometry description and gain insights into the origin of the observed CD signal inversions: i) *wrinkle direction angle*, to describe the angle of that wrinkle when visualized sideways or in cross‐section, and ii) *wrinkle orientation angle*, to describe the angle of that wrinkle when visualized in a front view through isosurface rendering (Figure 6b). The determined wrinkle direction angles for penta‐twinned and single‐crystalline dissymmetric products were ≈±25–30° (Figure 6c–i) and nearly 0° (Figure 6e–i), respectively. Moreover, the wrinkle orientations of these products were also found to be different, penta‐twinned and single‐crystalline wrinkled NRs displaying mainly negative and positive orientation angles, respectively (Figure 6d,f). In addition, the polar plots suggested that the highly complicated dissymmetric products partly and statistically preserved the rotational symmetries of penta‐twinned (C5) and single‐crystalline (C4) seeds. The noticed CD signal inversion effect as a function of seed crystal habit was also observed when employing the chemisorption method, advocating for further exploration of the growth mechanisms.^[^ ^81^ ^]^

**Figure 7:**
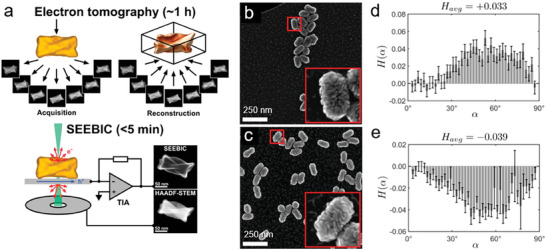
a) Schematic comparison of the working principles between electron tomography and SEEBIC. b,c) SEEBIC images of ***S***‐ and ***R***‐BINAMINE induced chiral Au NRs with helical wrinkles, respectively. d) Helicity quantification of an ***S***‐BINAMINE induced chiral Au NR ensemble, based on 14 overview SEEBIC images; e) Helicity quantification of an ***R***‐BINAMINE induced chiral Au NR ensemble, based on 12 overview SEEBIC images. More than 100 particles in each case were analyzed, and the averaged helicity showed nearly mirrored statistics, which, on the one hand, confirmed that different enantiomers led to mirrored nanostructures and, on the other hand, supported the feasibility and advantages of the SEEBIC technique. Reproduced with permission from ref.[83] (Copyright 2024 American Chemical Society).

Despite the demonstrated advantages of electron tomography for characterizing morphological features in chiral Au NPs, it is a highly time‐consuming technique, and therefore, it is difficult to characterize a relatively large number of particles. For this reason, a recent alternative has been developed by the Bals group, which enables the investigation of NP morphologies with a significantly higher throughput: Secondary Electron Electron Beam Induced Current (SEEBIC) (**Figure** **7**a).^[^ ^82^ ^]^ SEEBIC combines the advantages of scanning transmission electron microscopy (high resolution) and scanning electron microscopy (3D information and a higher number of particles per analysis). This results in a statistically much larger sample of characterized NPs, enabling an improved connection between the local NP structure and the overall optical properties of a given sample batch (Figure 7b–e).^[^ ^83^ ^]^ The surface details of more than 100 dissymmetric NRs can be captured in a few hours, and the helicities of these particles can be calculated accordingly.

## 6 Conclusions and Outlook

Wet‐chemical seed‐mediated growth routes have demonstrated unparalleled control over the synthesis of dissymmetric Au NRs. By spatiotemporal separation of nucleation, symmetry breaking, anisotropic growth, and chiral growth stages, colloidal approaches allow us to finely tune NP dimensions, crystal habit, and morphology. The formation of helical features or thin wrinkles is fundamental to obtaining NPs with strong chiroptical activity, whereas their quantitative relationships with chirality measures require further investigation. Kinetic conditions are needed to initiate the emergence of chiral features and support chiral growth, which requires the use of a large excess of ascorbic acid, a relatively low concentration of surfactants, and the presence of a chiral inducer. However, long‐term stabilization of such kinetic products also demands the presence of surface ligands that modify the NP's free surface energy and provide thermodynamic stability by decreasing the Gibbs surface energy of chiral NPs.

In all cases of chiral synthesis, the underlying mechanisms involve the removal of mirror planes present in the morphology of the employed seed particles. Excluding light‐induced chirality transfer from circularly polarized light to Au nanocolloids and focusing on the chemisorption‐directed growth strategy relies on thiolated chiral molecules, such as amino acids or peptides, to support the growth of dissymmetric protruding facets. This occurs by selective adsorption on chiral exposed planes, modifying their relative surface energies and providing thermodynamic stabilization. Notably, although cysteine and glutathione remain the most representative chiral shape‐inducing agents, many other chiral molecules, such as cysteine–phenylalanine^[^ ^6^, ^29^ ^]^ or adenine oligomers,^[^ ^84^ ^]^ have also shown great promise in inducing chiral Au NP growth. The second strategy, called micelle‐directed chiral growth, relies upon co‐surfactants that promote the formation of giant chiral micelles, which adsorb onto Au NRs and subsequently act as a template for the overgrowth of thin chiral wrinkles. Though initially investigated using BINOL and CTAC as the co‐surfactants, the use of BINAMINE and CTAC was found to be much more successful due to the presence of amine groups that improve micelle adsorption onto seed Au NRs.

Further advancements in the precision synthesis of chiral NR with desired shapes and sizes will undoubtedly require considering control over both the kinetics and thermodynamics of NR growth:

- Chiral‐shape directing agent: The discovery of effective chiral inducers holds both a notable challenge and a great potential for the development of novel chiral NPs. In this regard, a pre‐designed chiral inducer based on cysteine conjugated to a long aliphatic tail was recently reported to promote the growth of chiral NPs, either via chemisorption‐ or chiral micelle‐directed growth mechanism, depending on its concentration.^[^ ^35^ ^]^ Products were observed to range from a smooth and twisted geometry at low chiral inducer concentrations to one possessing well‐defined helical wrinkles at higher concentrations, suggesting changes in the chiral morphogenesis mechanisms. This work provides a promising route toward developing more efficient chiral molecules and further study inducer‐specific effects on chiral growth products.
- Reducing agent: The use of high [ascorbic acid]:[HAuCl_4_] ratios (above 25, i.e., closer to those used with the chiral micelle‐directed growth route) has a strong impact in the chemisorption‐directed growth strategy. It is also important to note that ascorbic acid is the reducing agent used in most cases for the growth of colloidal chiral NRs and NPs. However, other reducing agents, such as hydroquinone, have been used to grow Au NRs.^[^ ^41^, ^85^, ^86^ ^]^ Their oxidation potentials are lower than that of ascorbic acid, which often leads to slow growth kinetics. One can foresee their use at high concentrations to favor the kinetically controlled growth conditions required to form chiral NRs. In addition, reducing agents with distinct chemical nature may interact differently with Au NRs, potentially leading to dissymmetric growth pathways different from those observed in the case of ascorbic acid.
- pH: The growth kinetics is pH‐dependent. The oxidation potential of reducing agents such as ascorbic acid increases significantly at high pH, thereby enhancing their ability to reduce gold precursors.^[^ ^85^, ^87^ ^]^ Consequently, raising the pH in the chiral growth solution would potentially promote faster growth rates, thereby improving the chances of producing NPs with sharp chiral protruding facets and wrinkles. However, pH also determines the equilibrium between the protonated and deprotonated states of amino acid functional groups, which impacts their capacity to passivate gold surfaces: ‐SH > ‐S^−^, ‐NH_2_ > NH_3_^+^ and CO_2_^−^ > CO_2_H.^[^ ^88^ ^.^ ^90^ ^]^ The effect of pH in the growth of chiral Au NRs and NPs remains largely unexplored, representing a research direction with the potential to improve our understanding of chiral NP synthesis.
- Temperature: Most chiral NP growth methods are carried out at temperatures between RT and 30 °C. As discussed above, the growth of chiral protruding facets and wrinkles depends on the balance between gold atom precursor addition and surface diffusion rate. Although temperature affects both phenomena, it is often observed that anisotropy is favored under low‐temperature conditions.^[^ ^41^, ^91^ ^]^ One potential explanation would be that the decrease in the precursor reduction rate is less significant than surface diffusion. For these reasons, the growth of chiral NRs at temperatures well below RT could potentially enhance chiral growth.
- Surfactants and co‐surfactants: Surfactants play a crucial role in dissymmetric growth, where they exert thermodynamic (by passivating gold surfaces) and kinetic (by impacting the deposition of precursor atoms and complexing gold precursor) control.^[^ ^36^ ^]^ However, most chiral NP syntheses still rely on using CTAB and CTAC, with only few exceptions.^[^ ^28^, ^34^ ^]^ Different surfactants, such as benzyldimethylammonium chloride,^[^ ^92^ ^]^ cetylpyridinium chloride,^[^ ^93^ ^]^ or gemini surfactants (oligooxa) alkanedyl‐α,ω‐bis(dimethyldodecylammonium bromide^[^ ^94^ ^]^ have been successfully used in the growth of anisotropic Au NPs, often providing improved morphology control compared to CTAB or CTAC. Similarly, co‐surfactants such as sodium oleate^[^ ^95^ ^]^ or n‐decanol^[^ ^41^ ^]^ can enhance the shape‐directing properties of surfactants, yielding high‐quality anisotropic NPs. The implementation of well‐known surfactants and co‐surfactants, yet unexplored in chiral NR growth, may result in the fabrication of chiral NPs with unprecedented morphologies and stronger chiroptical activity.
- Alloys: Although we have focused our attention here on pure Au chiral NPs, exciting results have been recently obtained regarding the growth of chiral NP with more complex compositions. For example, AuAg nanorods with helical geometry can be prepared through overgrowth of Au NRs in the presence of silver ions and using cysteine as the chiral shape‐directing agent.^[^ ^96^ ^]^ Platinum wrinkles were also reported to form using the CTAC‐BINAMINE system.^[^ ^18^ ^]^ One could imagine exploring other plasmonic metals, such as copper, to produce alloy‐based chiral NPs with high optical activities and distinct chiral shapes.

During further exploration of different synthetic routes, advanced characterization and quantification of the dissymmetric and chiral 3D structure of the NP will remain essential. Such characterization will contribute to understanding chirality transfer from chiral inducers to nanoscale dissymmetric shapes and investigating the stability of the nanostructures, which can significantly propel the field of chiral synthesis. Moreover, a thorough structural characterization of the geometric features of NP is of great importance to structure‐property relations. The eventual development of electron tomography to map the local atomic handedness of NP surfaces (i.e., the absolute configuration) would be a powerful tool to fully understand which geometrical features determine the chirality of NPs. The SEEBIC technique opens another door for the fast determination of the geometric handedness, which could statistically provide the overall chirality of a synthesis batch. Other recent developments, such as the observation of ligands on NPs in liquid,^[^ ^97^ ^]^ may additionally provide evidence supporting one or the other growth mechanisms.

Starting from the pioneering work by Murphy and coworkers describing the seed‐mediated growth of gold NRs,^[^ ^98^ ^]^ this synthetic method has been adapted for shaping Au NPs into a myriad morphologies, and has recently become extremely convenient for the preparation of dissymmetric NPs. Novel applications based on these intricate structures would greatly fuel the field. To offer our view on the most promising directions, we should note that the functionality of chiral Au NPs can be ascribed to geometrical features and their optical response. Chirality in the atomic arrangement on crystal facets appears ideal for catalysts to enhance enantioselective chemical reactions. Moreover, we propose that chiral NP would display additional features, such as (i) tunable concentration of surface chiral centers, potentially allowing multi‐valent chiral binding, and (ii) chirality‐induced spin transfer effects capable of modulating catalytic selectivity. In the field of biomedicine, the intricate structures of chiral NPs have the potential to mimic the shape of proteins^[^ ^5^ ^]^ and other biomacromolecules.^[^ ^7^ ^]^ Therefore, strong binding between chiral NPs and certain proteins might be achieved, triggering different biological and immunological effects or showing unique biological activities.^[^ ^6^, ^99^, ^100^ ^]^ The optical properties of Au NPs could be exploited in this regard, meaning that preferential absorption of circularly polarized lights would result in selective heating for more accurate hyperthermia treatment or drug delivery.^[^ ^100^ ^]^ The development of chirality in the last centuries was accompanied by optical activities. However, the small molecular chirality has long gone beyond the scope of optics and has shown extremely important value in the field of life sciences. Now the chirality of inorganic materials is still mainly focused on optics, which on the other hand suggests there should be huge unexplored potential in other relevant fields as chirality is an important feature of nature.

## Conflict of Interest

The authors declare no conflict of interest.

## Supporting information



Supporting Information
