# Supplementary material for: Seed‐Mediated Growth and Advanced Characterization of Chiral Gold Nanorods
Source: Adv Mater. 2024 Oct 9;36(47):2412473. doi: 10.1002/adma.202412473 (PMC11586823; doi:10.1002/adma.202412473)
Supplement: Supplementary file 1 — Supporting Information [file ADMA-36-2412473-s001.docx]

Supporting Information

Seed-mediated Growth and Advanced Characterization of Chiral Gold Nanorods

Bing Ni*, Guillermo González-Rubio*, Kyle Van Gordon, Sara Bals, Nicholas A. Kotov, and Luis M. Liz-Marzán*

B. Ni, N. A. Kotov

Department of Chemical Engineering, University of Michigan, 2300 Hayward Street, Ann Arbor, Michigan 48109, United States

B. Ni,

Current Address: College of Chemistry, Beijing Normal University, 100875, Beijing, China

G. González-Rubio

Departamento de Química Física, Universidad Complutense de Madrid, Avenida Complutense s/n, 28040 Madrid, Spain

K. Van Gordon, L. M. Liz-Marzán

CIC biomaGUNE, Basque Research and Technology Alliance (BRTA), Paseo de Miramón 194, 20014 Donostia-San Sebastián, Spain

L. M. Liz-Marzán

Ikerbasque, Basque Foundation for Science, 48009 Bilbao, Spain

Biomedical Research Networking Center, Bioengineering, Biomaterials and Nanomedicine, CIBER-BBN, Paseo de Miramón 194, 20014 Donostia-San Sebastián, Spain

Cinbio, Universidade de Vigo, Campus Universitario s/n, 36310 Vigo, Spain

S. Bals

Electron Microscopy for Materials Science (EMAT) and NANOlab Center of Excellence, University of Antwerp, Groenenborgerlaan 171, 2020 Antwerp, Belgium

*E-mail: nib14@tsinghua.org.cn, ggrubio@ucm.es, llizmarzan@cicbiomagune.es

**Table S1. Some typical seed-mediated dissymmetric synthesis conditions of chiral Au NPs.**

(the cases of chiral Au NRs are highlighted in the table)

| **Chiral morphology** | **seed** | **Chiral inducer** | | **[HAuCl_4_] (mM)** | **[AA] (mM)** | **[AA] / [HAuCl_4_]** | **Surfactant** | | **Temp. (^o^C)** | **Maximal**  **\|g-factor\|** | **Note** | **Ref.** |
| --- | --- | --- | --- | --- | --- | --- | --- | --- | --- | --- | --- | --- |
|  |  | **Type** | **Conc. (μM)** |  |  |  | **Type** | **Conc. (mM)** |  |  |  |  |
| 432 helicoid I | Cube | Cys | 0.092 | 0.37 | 8.8 | 23.8 | CTAB | 15 | 30 | 0.03 (565 nm) |  | ^1^ |
| 432 helicoid II | Cube | GSH | 1.8 |  |  |  |  |  |  | 0.05 (575 nm) |  |  |
| 432 helicoid III | Octahedron | GSH | 4.6 |  |  |  |  |  |  | 0.2 (620 nm) |  |  |
| 432 helicoid IV | Rhombic dodecahedron | Cys | 0.094 | 0.19 | 8.9 | 47.5 | CTAB | 15 | 30 | 0.02 (~600nm) |  | ^2^ |
| 432 helicoid V | Octahedron | Cys-Gly | 0.14 |  |  |  | CTAB | 15 | 30 | 0.02 (620 nm) |  | ^3^ |
| Particle with rotation arms | octahedron | Adenine nucleobase | 0.05 |  |  |  | CTAB | 23 | 30 | 0.04 (~650nm) |  | ^4^ |
| 32 helicoid | Triangular plate | Cys | 4.7 |  |  |  | CTAB | 15 | 30 | 0.02 (1152 nm) | Sizes of seed can tune the morphology and optical properties | ^5^ |
| Particle with $\bar{4}3$ symmetry | Tetrahedron | GSH | 4.6 | 0.37 | 8.9 | 24.2 | CTAC/  CTAB | 5.9/  17.7 | 30 | ~0.05 (~600 nm) | Molar ratio between GSH to seed conc. is around 1.3~1.7 | ^6^ |
|  | Triangular plate |  |  |  |  |  | CTAB | 15 |  | ~0.015 (~550 nm) |  |  |
| Propeller-like particle | Octopod | Cys | 7.6 | 0.014 | 9.0 | 633 | CTAB | 15 | 30 | 0.002 (530 nm) | 8 HAuCl_4_ additions | ^7^ |
| Twisted Au nanorod | Single-crystalline rod | Cys | 0.24 | 0.014 | 9.0 | 633 | CTAB | 15 | 30 | 0.011 (550nm) | 8 HAuCl_4_ additions/﻿1-methylpyrrolidine added | ^8^ |
| Chiral trisoctahedron | sphere | Cys | 0.047 | 0.37 | 25.8 | 70 | CPC/  CPB | 33 | 30 | 0.02 (575 nm) | Ratio between Cys and seed conc. can greatly tune the morphology | ^9^ |
| Chiral particle with wrinkled surfaces |  |  | 0.47 |  |  |  |  |  |  | 0.0253 (645 nm) |  | ^10^ |
| Chiral truncated hexoctahedron and rhombic dodecahedron | Rhombicuboctahedron, octahedron, cube, rhombic dodecahedron, single-crystalline rod | CYP | 0.20 | 0.2 | 4.7 | 23.4 | CTAB | 16 | 30 | 0.01 (550 nm) | Optical activity inversion at different CYP conc., even though the same enantiomer is used | ^11^ |
| Chiral Au vortex particle | Rhombicuboctahedron, decahedron | GSH | 0.40 | 0.2 | 9.5 | 47.5 | CTAB | 16 | 30 | ~0.06 (600 nm) | 1μM of Cu(NO_3_)_2_ is added to modify the structure | ^12^ |
| 422 twisted rod | Single-crystalline rod | Cys | 0.074 | 0.0067 | 10.7 | 1600 | CTAC | 35.6 | 23 ~ 16 | 0.106 (650 nm) | 9 HAuCl_4_ additions at varied temperature to enhance the chirality | ^13^ |
| Nanotriskelion | Triangular plate or disk | GSH | 33 | 0.4 | 10 | 25 | CTAB | Varied | 35 | 0.12 (616 nm) | KI is added to modify the chiral morphology | ^14^ |
| Particles with 5-fold rotational symmetry | Decahedron, elongated decahedron, bipyramid | GSH | Varied | 0.63 | 3.1 | 5 | CTAB | 16 | 60 | ~ 0.06  (~ 650 nm) | 0.27 μM of KI | ^15^ |
| Nanopropeller | Decahedron | GSH | 3.6 | 0.37 | 8.8 | 23.8 | CTAB | 15 | 30 | 0.012 (697 nm) | Conc. of seeds is changed to obtain chiral structures with different features | ^16^ |
| Rod with spiral wrinkles | Single-crystalline rod | BIAMINE | 2500 | 0.5 | 158 | 317 | CTAC | 20 | r.t. | 0.2 (1100 nm, 1400 nm) | Chiral inducer serves as a co-surfactant | ^17^ |
| 422 twisted rod | Single-crystalline rod | LipoCYS | 20 | 0.19 | 700 | 3684 | CTAC | 44 | 40 | 0.025 (620 nm) | Conc. of chiral inducer can tune the growth mode | ^18^ |
| Twisted rod with wrinkles |  |  | 45 |  |  |  |  |  |  | 0.015 (700 nm) |  |  |
| Rod with spiral wrinkles |  |  | 90 |  |  |  |  |  |  | 0.066 (720 nm) |  |  |
| Propeller-like chiral particle | Triangular plate | CYP | 3.7 | 0.37 | 3.5 | 9.5 | CTAB | 1.5 | r.t. | 0.44 (727 nm) | Circularly polarized light is introduced to assist the dissymmetric growth | ^19^ |

AA: ascorbic acid; Cys: cysteine; GSH: glutathione; CYP: cysteine–phenylalanine, BIAMINE: 1,1′-binaphthyl-2,2′-diamine; LipoCYS: 2-amino-N-decyl-3-mercaptopropanamide;

CTAC: cetrimonium chloride; CTAB: cetrimonium bromide; CPC: cetylpyridinium Chloride; CPB: cetylpyridinium bromide

(1) H.-E- Lee, H.-Y. Ahn, J. Mun, Y. Y. Lee, M. Kim, N. H. Cho, K. Chang, W. S. Kim, J. Rho, K. T. Nam, Amino-acid- and peptide-directed synthesis of chiral plasmonic gold nanoparticles. *Nature* **2018**, *556*, 360-365.

(2) H.-E. Lee, R. M. Kim, H.-Y. Ahn, Y. Y. Lee, G. H. Byun, S. W. Im, J. Mun, J. Rho, K. T. Nam, Cysteine-encoded chirality evolution in plasmonic rhombic dodecahedral gold nanoparticles. *Nature Communications* **2020**, *11*, 263.

(3) H. Kim, S. W. Im, N. H. Cho, D. H. Seo, R. M. Kim, Y.-C. Lim, H.-E. Lee, H.-Y. Ahn, K. T. Nam, γ-Glutamylcysteine- and Cysteinylglycine-Directed Growth of Chiral Gold Nanoparticles and their Crystallographic Analysis. *Angew. Chem. Int. Ed.* **2020**, *59*, 12976-12983.

(4) N. H. Cho, Y. B. Kim, Y. Y. Lee, S. W. Im, R. M. Kim, J. W. Kim, S. D. Namgung, H.-E. Lee, H. Kim, J. H. Han, H. W. Chung, Y. H. Lee, J. W. Han, K. T. Nam, Adenine oligomer directed synthesis of chiral gold nanoparticles. *Nat. Commun.* **2022**, *13*, 3831.

(5) S. W. Im, E. Jo, R. M. Kim, J. H. Han, K. T. Nam, 32-Symmetric Chiral Gold Nanoplates with Near-Infrared Circular Dichroism. *Adv. Opt. Mater.* **2023**, *11*, 2300037.

(6) J. S. Googasian, G. R. Lewis, Z. J. Woessner, E. Ringe, S. E. Skrabalak, Seed-directed synthesis of chiroptically active Au nanocrystals of varied symmetries. *Chem. Commun.* **2022**, *58*, 11575-11578.

(7) N.-N. Zhang, H.-R. Sun, Y. Xue, F. Peng, K. Liu, Tuning the Chiral Morphology of Gold Nanoparticles with Oligomeric Gold–Glutathione Complexes. *J. Phys. Chem. C* **2021**, *125*, 10708-10715.

(8) N.-N. Zhang, Z.-L. Shen, S.-Y. Gao, F. Peng, Z.-J. Cao, Y. Wang, Z. Wang, W. Zhang, Y. Yang, K. Liu, T. Sun, Synthesis and Plasmonic Chiroptical Properties of Double-Helical Gold Nanorod Enantiomers. *Adv. Opt. Mater.* **2023**, *11*, 2203119.

(9) F. Wu, Y. Tian, X. Luan, X. Lv, F. Li, G. Xu, W. Niu, Synthesis of Chiral Au Nanocrystals with Precise Homochiral Facets for Enantioselective Surface Chemistry. *Nano Lett.* **2022**, *22*, 2915-2922.

(10) F. Wu, F. Li, Y. Tian, X. Lv, X. Luan, G. Xu, W. Niu, Surface Topographical Engineering of Chiral Au Nanocrystals with Chiral Hot Spots for Plasmon-Enhanced Chiral Discrimination. *Nano Lett.* **2023**, *23*, 8233-8240.

(11) X. Sun, J. Yang, L. Sun, G. Yang, C. Liu, Y. Tao, Q. Cheng, C. Wang, H. Xu, Q. Zhang, Tunable Reversal of Circular Dichroism in the Seed-Mediated Growth of Bichiral Plasmonic Nanoparticles. *ACS Nano* **2022**, *16*, 19174-19186.

(12) J. Wan, L. Sun, X. Sun, C. Liu, G. Yang, B. Zhang, Y. Tao, Y. Yang, Q. Zhang, Cu^2+^-Dominated Chirality Transfer from Chiral Molecules to Concave Chiral Au Nanoparticles. *J. Am. Chem. Soc.* **2024**, *146*, 10640-10654.

(13) B. Ni, M. Mychinko, S. E. Gómez-Graña, J. Morales-Vidal, M. Obelleiro-Liz, W. Heyvaert, D. Vila-Liarte, X. Zhuo, W. Albrecht, G. Zheng, G. González-Rubio, J. M. Taboada, F. Obelleiro, N. López, J. Pérez-Juste, I. Pastoriza-Santos, H. Cölfen, S. Bals, L. M. Liz-Marzán, Chiral Seeded Growth of Gold Nanorods Into Fourfold Twisted Nanoparticles with Plasmonic Optical Activity. *Adv. Mater.* **2023**, *35*, 2208299.

(14) J. Zheng, C. Boukouvala, G. R. Lewis, Y. Ma, Y. Chen, E. Ringe, L. Shao, Z. Huang, J. Wang, Halide-Assisted Differential Growth of Chiral Nanoparticles with Threefold Rotational Symmetry. *Nat. Commun.* **2023**, *14*, 3783.

(15) L. Zhang, Y. Chen, J. Zheng, G. R. Lewis, X. Xia, E. Ringe, W. Zhang, J. Wang, Chiral Gold Nanorods with Five-Fold Rotational Symmetry and Orientation-Dependent Chiroptical Properties of Their Monomers and Dimers. *Angew. Chem. Int. Ed.* **2023**, *62*, e202312615.

(16) Y. Zheng, X. Li, L. Huang, X. Li, S. Yang, Q. Wang, J. Du, Y. Wang, W. Ding, B. Gao, H. Chen, Homochiral Nanopropeller via Chiral Active Surface Growth. *J. Am. Chem. Soc.* **2024**, *146*, 410-418.

(17) G. González-Rubio, J. Mosquera, V. Kumar, A. Pedrazo-Tardajos, P. Llombart, D. M. Solís, I. Lobato, E. G. Noya, A. Guerrero-Martínez, J. M. Taboada, F. Obelleiro, L. G. MacDowell, S. Bals, L. M. Liz-Marzán, Micelle-Directed Chiral Seeded Growth on Anisotropic Gold Nanocrystals. *Science* **2020**, *368*, 1472-1477.

(18) K. Van Gordon, S. Baúlde, M. Mychinko, W. Heyvaert, M. Obelleiro-Liz, A. Criado, S. Bals, L. M. Liz-Marzán, J. Mosquera, Tuning the Growth of Chiral Gold Nanoparticles Through Rational Design of a Chiral Molecular Inducer. *Nano Lett.* **2023**, *23*, 9880-9886.

(19) L. Xu, X. Wang, W. Wang, M. Sun, W. J. Choi, J.-Y. Kim, C. Hao, S. Li, A. Qu, M. Lu, X. Wu, F. M. Colombari, W. R. Gomes, A. L. Blanco, A. F. de Moura, X. Guo, H. Kuang, N. A. Kotov,C. Xu, C. Enantiomer-Dependent Immunological Response to Chiral Nanoparticles. *Nature* **2022**, *601*, 366-373.
